# Development and Clinical Application of a Multilocus Sequence Typing Scheme for *Bacteroides fragilis* Based on Whole-Genome Sequencing Data

**DOI:** 10.1128/spectrum.05111-22

**Published:** 2023-03-21

**Authors:** Flemming D. Nielsen, Marianne N. Skov, Thomas V. Sydenham, Ulrik S. Justesen

**Affiliations:** a Department of Clinical Microbiology, Odense University Hospital, Odense, Denmark; b Research Unit of Clinical Microbiology, Department of Clinical Research, University of Southern Denmark, Odense, Denmark; Nevada State Public Health Laboratory

**Keywords:** *Bacteroides fragilis*, MLST, genome typing, whole-genome sequencing, population structure, carbapenemase gene (*cfiA*), anaerobes, antibiotic resistance, bacteremia, bioinformatics, genomics, pathogenesis

## Abstract

Bacteroides fragilis is among the most abundant and pathogenic bacterial species in the gut microbiota and is associated with diarrheal disease in children, inflammatory bowel disease, and the development of colorectal cancer. It is increasingly resistant to potent antimicrobial agents such as carbapenems and metronidazole, making it among the most resistant anaerobic bacteria. These factors combined call for increased monitoring of B. fragilis and its population structure on a worldwide scale. Here, we present a possible solution through the development of a multilocus sequence typing scheme (MLST). The scheme is based on seven core gene fragments: *groL* (*hsp60*), *rpoB*, *recA*, *dnaJ*, *rprX*, *prfA*, and *fusA*. These gene fragments possess high discriminatory power while retaining concordance with whole core genome-based phylogenetic analysis. The scheme proved capable of differentiating B. fragilis isolates at the strain level. It offers a standardized method for molecular typing and can be applied to isolates from various sampling backgrounds, such as patient isolates, environmental samples, and strains obtained from food and animal sources. In total, 567 B. fragilis genomes were sequence typed and their isolate data collected. The MLST scheme clearly divided the B. fragilis population into two divisions based on the presence of the *cfiA* and *cepA* resistance genes. However, no other specific subpopulations within the analyzed genomes were found to be associated with any specific diseases or geographical location. With this MLST scheme, we hope to provide a powerful tool for the study and monitoring of B. fragilis on an international scale.

**IMPORTANCE** Here, we present the first MLST scheme for Bacteroides fragilis, one of the most abundant pathogenic bacteria in the human gut microbiota. The scheme enables standard classification and monitoring of B. fragilis on a worldwide scale and groups the majority of current isolate data in one place. A more unified approach to the collection and analysis of B. fragilis data could provide crucial insights into how the pathogen operates and develops as a species. Close monitoring of B. fragilis is especially relevant, as it is increasingly resistant to potent antimicrobial agents and engages in horizontal gene transfer with other bacteria. Hopefully, this approach will guide new discoveries into how B. fragilis evolves and interacts with its human host. Additionally, the scheme could potentially be applied to other species of the genus *Bacteroides*.

## INTRODUCTION

*Bacteroides* spp. are among the most abundant and pathogenic bacterial species in the gut microbiota. Bacteroides fragilis is the number one pathogen (“king of the colon”) within the genus *Bacteroides*, with the most cases of anaerobic bacteremia (1–3). B. fragilis is also among the most resistant anaerobic bacteria, and increasing resistance to potent antimicrobial agents such as carbapenems and metronidazole has been reported worldwide (1). Resistance to carbapenems and metronidazole is typically, but not always, mediated by or associated with the *cfiA* and *nim* genes, respectively (4). B. fragilis has also been associated with diarrheal disease in children, inflammatory bowel disease, and the development of colorectal cancer caused by the B. fragilis toxin (BFT) (5–7).

Based on these different characteristics and associations, many studies have investigated the B. fragilis genomic background and phylogeny, e.g., the distribution of the *cfiA* and the *bft* genes, to evaluate the genetic relationship between strains (5, 8, 9). It is clear from these studies that B. fragilis can be divided into two divisions, I and II, based on DNA-DNA hybridization. The two divisions can be identified based on the absence (division I) or presence (division II) of the metallo-β-lactamase-encoding gene *cfiA* (9). Additionally, division I *B. fragilis* will often have the resistance gene *cepA*, a cephalosporinase, whereas division II bacteria have not been found to carry this gene. Although some genes can be associated with certain genotypes, studies have not demonstrated clonal relatedness between isolates from closely related sources, e.g., children or hospitals, and resistance or specific diseases or pathogenic properties.

To our knowledge, a multilocus sequence typing (MLST) approach has not been used to investigate these types of relationships before in Bacteroides fragilis. MLST provides a standardized classification method that may serve as a framework for global phylogenetic investigations. The typical MLST scheme is based on 6 to 10 internal fragments of known housekeeping genes, making this classification method highly accessible and easy to reproduce (10).

The primary purposes of this study were (i) to develop a MLST scheme for B. fragilis based on whole-genome sequencing (WGS) data and (ii) to use this scheme to look for MLST differences between the strains causing bacteremia, from different sources (such as animals versus humans), displaying resistance, and harboring the B. fragilis toxin and to look for possible outbreaks and geographical variation. A secondary purpose was to examine the applicability of the scheme to other species belonging to the genus *Bacteroides*.

## RESULTS

To identify suitable MLST gene candidates with high phylogenetic discriminatory power, a number of selection criteria were set. To be considered a candidate gene, the gene had to (i) be present in every isolate and only once; (ii) encode a product of known function; (iii) contain variable regions of polymorphic sites; and (iv) have conserved regions of suitable distance for fragment PCR amplification.

**FIG 1:**
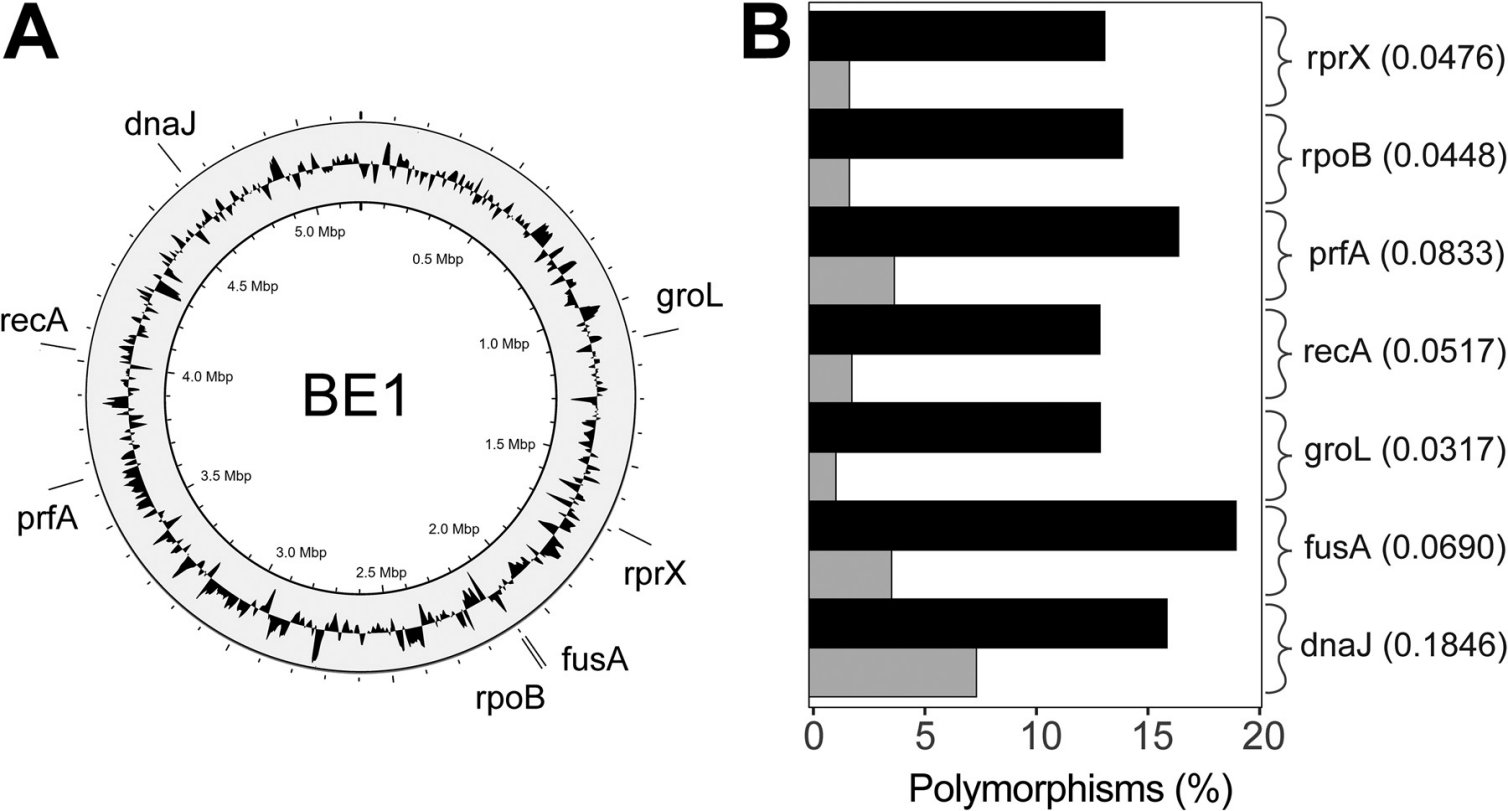
Distribution and genetic variation of the seven Bacteroides fragilis MLST loci. (A) Genomic position of the loci across the B. fragilis BE1 reference genome. (B) Percentage of polymorphic sites for each locus, with nucleotide polymorphisms in black and amino acid polymorphisms in gray. The ratio of nonsynonymous to synonymous mutations is listed to the right of each fragment.

To identify gene candidates, a pangenome was constructed based on a collection of 67 B. fragilis genomes, which consisted of 21,816 unique genes. Of these genes, 1,466 were present across all isolates, making them core genes. The core genome was then sorted to exclude any genes present more than once in any isolate. This identified 1,088 single-copy core genes as possible MLST gene candidates. Of the core genes, 15 candidate genes were chosen based on their function and documentation in the literature. The genes *groL* (*hsp60*), *rpoB*, *gyrB*, *dnaJ*, and *recA* have previously been shown to display good phylogenetic discriminatory power in *Bacteroides* due to their high intraspecies sequence variation (11, 12). The genes *ftsH*, *fusA*, *infA*, *infB*, *nusG*, *polA*, *prfA*, *rprX*, *ruvB*, and *tuf* are known to play key biological roles in B. fragilis, are essential for survival, and are homologs to many of the genes that are used in MLST schemes for other species (13). Each candidate gene was then assessed across all genomes for variable regions of ~500 bp, flanked by conserved regions suitable for primer design. Of the genes analyzed, the seven loci that seemingly had the best discriminatory power, while still being suitable for PCR amplification, were *groL*, *rpoB*, *recA*, *dnaJ*, *rprX*, *prfA*, and *fusA* (Fig. 1A and Table 1).

**TABLE 1:** Features of the seven loci of the Bacteroides fragilis MLST scheme

Locus	Gene product	Length (bp)	Position^*a*^	No. of alleles	No. of polymorphic sites	No. of nonsynonymous mutations
*rprX*	Histidine kinase	498	4278139	31	66	3
*rpoB*	RNA polymerase b-subunit	498	4701365	23	70	3
*prfA*	Release factor	471	1062510	50	78	6
*recA*	DNA repair recombinase	468	1436330	28	61	3
*groL*	Heat shock protein	498	3715200	28	65	2
*fusA*	Elongation factor G	486	4689327	44	93	6
*dnaJ*	Chaperone protein	480	2039111	51	77	12

a The start position of each fragment is listed according to its position within the B. fragilis BE1 reference genome.

Primer sequences and MLST amplicon information are provided in Table S1 in the supplemental material.

### Sequence variation within loci.

The seven loci were selected for further analysis, which was expanded to include 567 B. fragilis genomes. In total, 255 unique allele sequences were identified across the seven loci. The number of polymorphic sites in each allele ranged from 61 in *recA* to 93 in *fusA* (Table 1). Overall, the concatenated length of the loci was 3,399 bp, of which 510 bp (15%) were variable.

The degree of selective pressure on each gene fragment was assessed by calculating the ratio of nonsynonymous to synonymous mutations (*K_A_*/*K_S_*). If this ratio is less than 1, the gene is expected to undergo purifying selection. The ratio was found to range from 0.0317 in *groL* to 0.1846 in *dnaJ*, implying purifying selection for all fragments. This indicates that the fragments are genetically stable and that alterations at the amino acid level are strongly selected against (Fig. 1B).

To assess the phylogenetic relevance of the alleles at different loci, the standardized index of association (I_A_^S^) was calculated. If alleles are freely distributed among the population through genetic recombination, then the I_A_^S^ value is expected to be zero. However, if the I_A_^S^ value is not equal to zero, there is a linkage disequilibrium, as the alleles are nonrandomly associated at different loci. The I_A_^S^ value in this study was found to be 0.1999, indicating that the alleles at different loci were predominantly a product of clonality (14).

### Sequence type assignment.

The 567 isolates were typed and distributed into 202 unique sequence types (STs), numbered according to their gradual identification. In total, 96 STs had more than one associated isolate, and 106 STs were associated with a single isolate. The ST associated with the most isolates was ST77, which contained 42 isolates.

In total, 133 of the isolates were confirmed as *cfiA* positive and were assigned to division II. Isolates of the same ST always belonged to the same division. A phylogenetic analysis was performed on the concatenated MLST fragments, and the STs formed two distinct groups, according to their assigned division.

**FIG 2:**
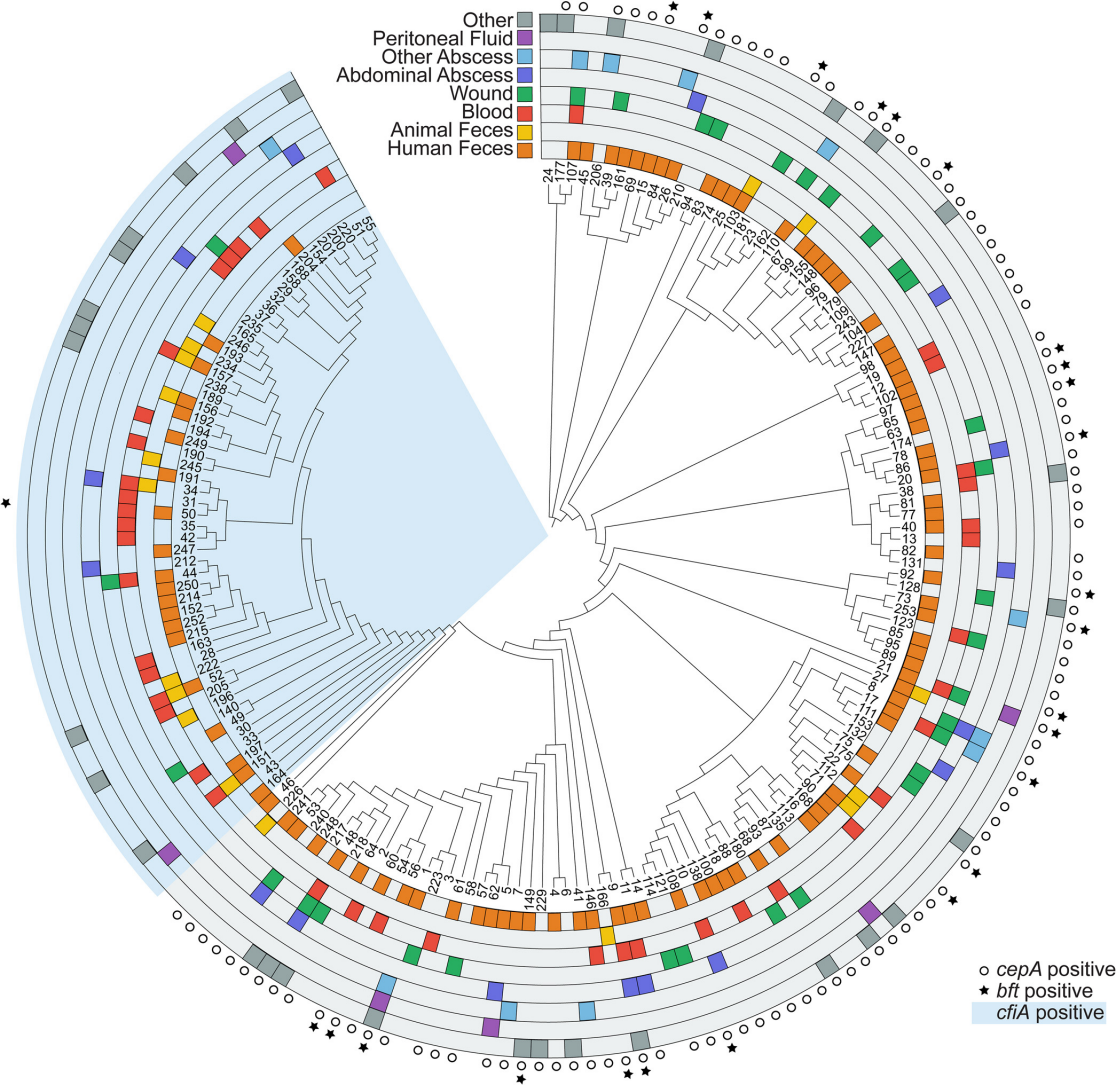
Unrooted phylogenetic tree of the identified sequence types (STs) based on the concatenated MLST fragments. Branch lengths are not to scale. The isolation sources are indicted in the outer rings of the tree. The STs associated with division II are highlighted in light blue. STs for which one or more isolate carried the genes *bft* or *cepA* are marked.

The *cepA* gene was only found in division I genomes, but not in all of them (89%). The *bft* gene was predominantly found in division I genomes, except for a single division II isolate harboring the *bft-2* gene (Fig. 2). Overall, most STs were isolated from feces (56%). Division II STs were isolated from blood cultures twice as often as division I isolates (36% versus 18%) (Fig. 2).

### Clonal complexes.

A total of eight clonal complexes (CCs) and 22 singletons were defined. The size of the clonal complexes varied from 115 STs in CC1 to 2 STs in CC8. All CCs consisted of STs belonging to either division I or II exclusively. CC1 and CC2 consisted of STs associated with division I, and CC3 to CC8 consisted of STs associated with division II. Of the singletons, 18 were associated with division II, whereas only four singletons were associated with division I.

Potential ancestral types were also identified for CCs if one ST was linked to a significantly greater number of others STs through single locus variations. ST112 was categorized as an ancestral type for CC1 and ST55 for CC5.

**FIG 3:**
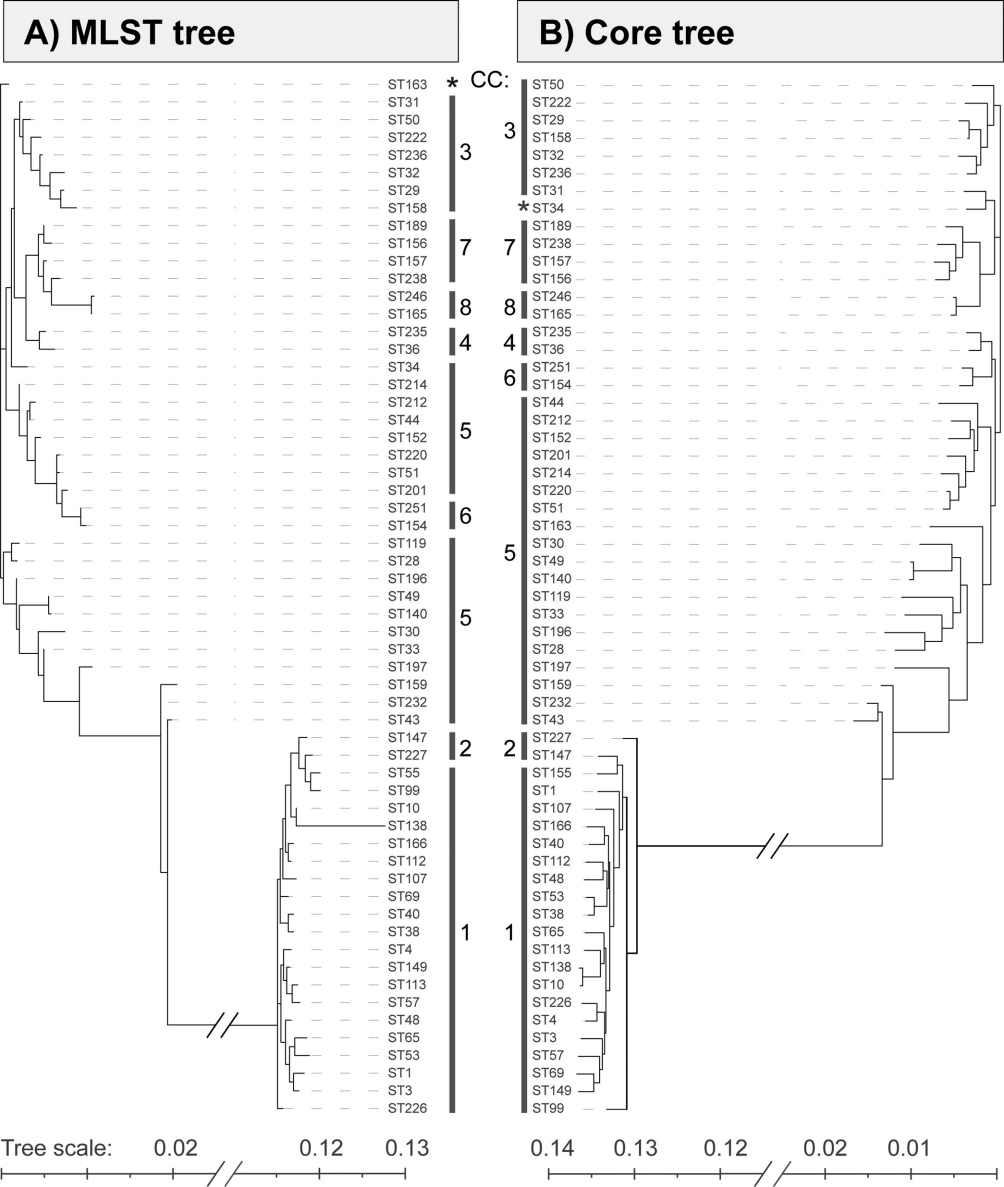
Phylogenetic trees based on the concatenated MLST fragments of sequence type (ST) (A) and single nucleotide polymorphisms within the core genome (B). Tree distances are to scale, with a portion of the tree separating the two genogroups removed as indicated by the line breaks. Representative genomes were picked for each ST, except for CC1, for which the number was limited to 20 unique STs chosen at random. Node labels represent STs, and the flanking lines indicate the accommodating clonal complex (CCs). *, STs that belonged to CC5 but only appeared in the associated cluster in one of the trees.

To evaluate the phylogenetic accuracy of the MLST scheme, it was compared to a single nucleotide polymorphism (SNP)-based phylogenetic analysis of the entire core genome. Representative genomes were picked for each ST, except for CC1, for which the number was limited to 20 unique STs chosen at random. The MLST tree was based on the concatenated sequences of each ST, whereas the core genome tree was based on SNPs across 1,177 core genes (Fig. 3). Both phylogenetic analyses clustered the STs according to the assigned CCs and displayed the concordance between the whole core genome phylogeny and the designed MLST scheme. In both cases, CC6 displayed a closer phylogenetic relationship to satellites of CC5 than some STs of CC5 themselves; however, CC6 still failed to have four or more alleles in common with any member of CC5.

Additionally, ST163 and ST34 only clustered with their associated CC5 STs in one of the phylogenetic analyses. Overall, the MLST scheme displayed concordant clustering according to clonal complexes, compared to the complete core genome SNP-based phylogenetic tree.

**FIG 4:**
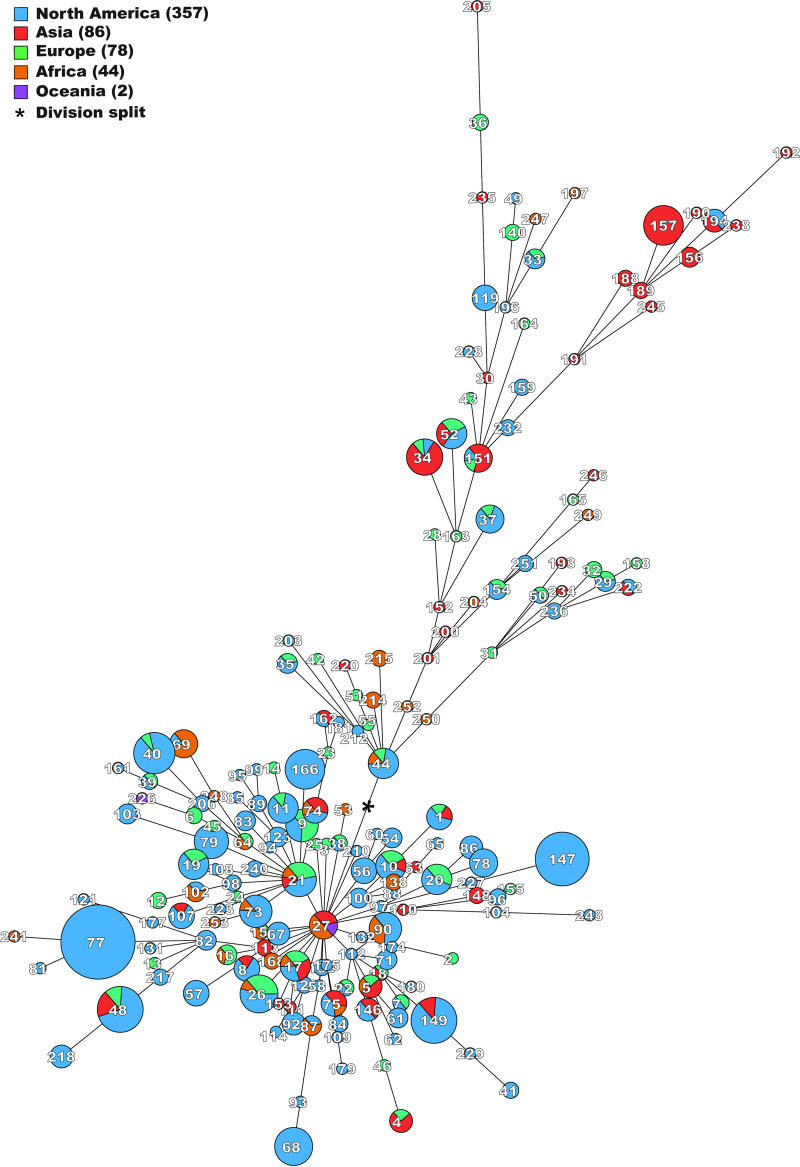
Grapetree diagram based on the sequence types (STs) of Bacteroides fragilis. Nodes are scaled according to the number of isolates associated with each ST. Node colors indicate in which continents each ST has been isolated. The number in each circle indicates the sum of isolates from all continents. The tree splits between divisions I and II at the asterisk, with the lower part representing division I and the upper part division II.

Most division I isolates were submitted from North America (72%), and division II isolates were predominantly submitted from Asia (38%). One division II cluster in particular was associated with Asia and consisted of CC7 and its satellites (Fig. 4).

Finally, the potential of the MLST scheme to classify other species within the genus *Bacteroides* was assessed. This was achieved by screening for the complete sequence of the seven loci in a representative genome from each of the species: B. thetaiotaomicron, B. ovatus, B. uniformis, and B. intestinalis. It was found that these species indeed possess the complete sequence of the seven loci and could therefore be classified using this MLST scheme (12). All of the genomes from these species were assigned new non-B. fragilis STs. The scheme was also tested on a former member of the *Bacteroides* genus, Phocaeicola vulgatus; however, it did not possess a close homolog to the *rprX* gene.

## DISCUSSION

A multilocus sequence typing (MLST) scheme was developed for Bacteroides fragilis. The scheme was based on internal fragments from seven single-copy core genes associated with housekeeping functions. The genes selected for each locus are *groL*, *rpoB*, *recA*, *dnaJ*, *rprX*, *prfA*, and *fusA*.

To accommodate the scheme, a PubMLST database containing all the sequence typing and isolate data was created (https://pubmlst.org/organisms/bacteroides-fragilis). The database is set up for researchers to conveniently submit their own typing data, as well as to perform in-depth phylogenetic analyses using the existing typing data. Analyses include, but are not limited to, the phylogenetic relatedness of strains, sequence type alignments, and SNP tracking, as well as linking to the extensive metadata of both isolate pathology and geography.

Isolates from bacteremia included many different STs, with no distinct pattern. There were several bacteremia isolates allocated to division II (*cfiA* positive). This is believed to have been caused by selection bias because division II isolates were more resistant to carbapenems and were probably selected for sequencing based on this feature and the seriousness of the infection.

Single STs were widely dispersed across multiple sampling sites and sources. Two examples are the division II isolates BF_BC_OUH_DK_2010_18 (ST34) and BF_BC_OUH_DK_2010_85 (ST29), both of which were isolated at Odense University Hospital in 2010 (see Table S3 in the supplemental material). Interestingly, isolate BF_BC_OUH_DK_2010_18 shared a closer phylogenetic relationship to seven B. fragilis isolates from Hong Kong that were isolated from chicken feces in 2017. However, both isolates belonged to CC5. Other animal fecal samples often shared an ST with human fecal isolates; however, this was mostly true for the primate-sourced samples. This phenomenon could be due to the “humanization” of animal gut microbiomes. It has been documented that both domesticated and wild animals living in proximity to urban environments acquire gut microbiota constituents found in humans, including *Bacteroides* spp. (15, 16).

Several of the isolates were from Odense University Hospital (*n* = 19) and from a short period of time (2006 to 2016), but in spite of this, no outbreaks or possible transmission between patients could be detected based on the data. To our knowledge, hospital outbreaks of B. fragilis have not been reported; however, we do not know whether this has been evaluated systematically before. Apparently, B. fragilis can spread among primates from the same enclosure, as seen with the 11 isolates constituting ST166, but it is expected that simple hygiene and infection control measures at a hospital will prevent this in humans. Furthermore, as B. fragilis is an anaerobic bacteria, survival outside the host will probably be limited, reducing the transmission potential.

STs from all over the world grouped together, apart from some STs belonging to division II and allocated almost solely to Asia (Fig. 4). It was expected that some degree of geographical clustering would be present; instead, such clustering was more of an exception. The MLST data also provided no evidence that any specific subpopulations were responsible for the various diseases caused by B. fragilis alone. This is not uncommon and is also seen with other similar MLST schemes, such as those for Staphylococcus aureus and Streptococcus pyogenes, both of which are still in use (17, 18). However, this could indicate that potential virulence genes are obtained through recombination rather than through clonality.

The distribution of the two resistance genes *cfiA* and *cepA* was confirmed, as all division II isolates had the gene *cfiA*, whereas many division I isolates had the *cepA* gene, but no association with STs could be detected. Similarly, the distribution of the *bft* gene was apparently random within division I; interestingly, however, only one isolate from division II was found to carry the *bft* gene.

Overall, the MLST scheme developed provides an accessible classification method for B. fragilis isolates. The typing scheme proved capable of analyzing B. fragilis isolates from various sampling backgrounds, such as patient isolates, environmental samples, and strains obtained from food and animal sources. The scheme also displays high discriminatory power down to the strain level, while retaining good concordance with the whole core genome-based phylogenetic analysis. As the high discriminatory power seen in whole core genome-based analysis is retained by using only seven gene fragments, phylogenetic analyses can be performed with only a fraction of the computational requirements. Additionally, MLST typing, compared to whole-genome phylogeny, is stable over time and not subject to major changes as new isolates are sequenced; this makes the method backward compatible and enables epidemiologists to compare results. With this MLST scheme, we hope to provide a growing resource which may be used to address the population and evolutionary aspects of the species on an international scale.

## MATERIALS AND METHODS

A detailed overview of all software programs and packages used for this study can be found in Table S2 in the supplemental material.

### Genomes.

To identify single-copy core genes, a collection of 67 B. fragilis genomes were used. Of these genomes, 48 were sequenced in-house for use in this study. These isolates were selected for sequencing based on diversity to secure a broad spectrum, including fecal isolates from children and adults and isolates from bacteremia, different countries, food, and from different periods of time. The remaining 19 genomes were retrieved from the National Center for Biotechnology Information (NCBI; accessed September 2021). We specifically chose to retrieve only complete assemblies from NCBI to reduce the risk of missing core genes due to sequencing errors. In total, 19 of the genomes were confirmed *cfiA* positive.

To test the selected loci and the MLST scheme itself, the analysis was expanded to include all available genome sequences of the species available from NCBI (as of June 2022). In total, complete MLST profiles were successfully extracted from 567 B. fragilis genomes. A list of the genomes is available in Table S3 in the supplemental material.

### Whole-genome sequencing and *de novo* assembly.

The genomes sequenced in-house for use in this study were sequenced with paired-end short-read sequencing, using the Illumina MiSeq platform. Genomic DNA was purified using the MasterPure DNA purification kit (Epicentre Biotechnologies, Madison, WI, USA), and libraries were prepared using the Nextera XT kit (Illumina, Essex, UK), both following the standard protocol of the manufacturers.

The quality of the WGS data was determined using FastQC, and the read coverage was calculated using the Lander/Waterman equation with a cutoff of 36× coverage (19, 20). Data of sufficient quality were assembled using the SPAdes-based pipeline Shovill, with default settings (21).

### Pangenome assembly and analysis.

A pangenome was created using the 67 unique B. fragilis genomes, as previously described. To ensure consistent gene identification, all genomes were annotated using Prokka with default settings (22). The pangenome was then assembled from the annotated genomes using Roary with the following settings: -e (multiFASTA alignment of core genes), -s (group paralogs), -ap (allow paralogs in core), -cd 100 (core genes must be in 100% of genomes), -i 95 (genes must have 95% identity using blastp) (23). As the core genome was identified as genes present in 100% of genomes, only complete assemblies were retrieved from GenBank to ensure that no true core genes were missing due to poor coverage.

### MLST locus selection.

Suitable MLST loci were selected according to the guidelines provided by Maiden, with some modifications (24). The sequence of each candidate gene was then extracted from each isolate, and a multiple sequence alignment (MSA) was performed using the Clustal algorithm (25). Based on the MSA, genes with variable regions flanked by conserved regions suitable for primer adherence were selected.

Primers were designed with the aim of achieving a GC content of 40% to 60% and a melting temperature (*T_m_*) of ~55°C with MLST fragments of ~500 bp and an ~50-bp flanking region on either side of the amplicon to accommodate Sanger sequencing. To enable convenient translation to amino acid sequences, the fragments were to encompass only complete operons.

### Phylogenetic trees.

Core genome-based phylogenetic trees were created by running Roary on the selected genomes as previously described. The resulting multiFASTA alignment of the core genome was used as an input for FastTree to create an unrooted phylogenetic tree, based on SNPs (26). FastTree was used with default settings.

MLST-based phylogenetic trees were created by performing a MSA on the concatenated MLST fragments from each isolate. The fragments, totaling 3,399 bp, were concatenated in the following order: *rprX*, *rpoB*, *prfA*, *recA*, *groL*, *fusA*, and *dnaJ*. The MSA was performed using ClustalW through the msa package in R with default settings (25). The resulting MSA was used as an input for FastTree as previously described.

The phylogenetic trees were illustrated using the Interactive Tree of Life (iTOL) and exported to Adobe Illustrator (27).

### MLST locus analysis.

The numbers of synonymous and nonsynonymous mutations were determined using the Locus Explorer analysis tool on PubMLST (28), and the ratio of nonsynonymous to synonymous mutations (K_A_/K_S_) was then determined. The standardized index of association was calculated using LIAN 3.7 (29) and was defined as follows:

$$I_{A}^{\;s}=\frac{1}{l-1}\left(\frac{V_{D}}{V_{e}}-1\right)$$

### Identification of clonal complexes.

Clonal complexes were identified using goeBURST (30). The software was used with default settings and with a group definition of *n* − 3 profile match, with *n* being the total number of alleles in the scheme.

### Gene screening across isolates.

Reference sequences of the *cfiA*, *cepA*, and *bft* genes were retrieved from the RefSeq nucleotide archive and translated to amino acid sequences using the ExPASy translation tool (31). A tBLASTn search was performed for each reference sequence against all genomes using BLAST+ (32). An E value cutoff of 10E–50 was chosen to only include nearly identical matches.

### MLST locus assessment in other *Bacteroides* species.

To test whether the MLST scheme had the potential to classify isolates from other members of the genus *Bacteroides*, a number of representative genomes were selected. The representative genomes selected were B. thetaiotaomicron VPI-5482, *B. ovatus* ATCC 8483, B. uniformis NBRC 113350, *B. intestinalis* APC919/174, and *Phocaeicola vulgatus* ATCC 8482 (previously B. vulgatus). These strains were specifically chosen as they belong to different clades within the genus *Bacteroides*. The amino acid sequences of each MLST fragment were subjected to a BLAST search against each reference genome with an E value cutoff of 10E–50 for identical matches. The output was manually assessed for sequence length, identity, and potential paralogs that might interfere with the sequence typing.

### Data availability.

The genomes sequenced for this study have been deposited at GenBank under BioProject accession number PRJNA910333.
