# Supplementary material for: Development and Clinical Application of a Multilocus Sequence Typing Scheme for Bacteroides fragilis Based on Whole-Genome Sequencing Data
Source: Microbiol Spectr. 2023 Mar 21;11(2):e05111-22. doi: 10.1128/spectrum.05111-22 (PMC10101032; doi:10.1128/spectrum.05111-22)
Supplement: Supplemental file 1 — Tables S1 and S2. Download spectrum.05111-22-s0002.pdf, PDF file, 0.2 MB [file spectrum.05111-22-s0002.pdf]

Table S1. The Bacteroides fragilis MLST scheme uses internal fragments of seven single-copy core genes associated with housekeeping functions. The fragments are amplified using the following primers pairs and PCR protocol.

Primer	Sequence (5´ to 3´)	Amplicon size (bp)	MLST fragment size (bp)
**GroL - Heat shock protein**			
groL_fw	CGGTTATCGGTAAACTGATTGC	607	498
groL_rv	GATTTAGTAGCAGCAATCTGAGC		
**RpoB - RNA polymerase b-subunit**			
rpoB_fw	GCCGATTATCCGGTTGTAG	614	498
rpoB_rv	CGAACTTCGAGTGAATACTCTTCTAC		
**DnaJ - Chaperone protein**			
dnaJ_fw	GGATAAACGTGCCCGCTAC	582	480
dnaJ_rv	C(G/C)CCCATAGAGAGTTGC		
**RprX - Histidine kinase**			
rprX_fw	TACATCCGTGCGAAATGC	607	498
rprX_rv	CTTCACAATACTCATCTTCGCAG		
**PrfA - Release factor**			
prfA_fw	CTCAGGA(T/C)GGTAA(G/A)AATGCC	573	471
prfA_rv	CGTCGATATACTTCTGATGTTCC		
**FusA- Elongation factor G**			
fusA_fw	CTACAACTCTCGTTCAGGTAAG	588	486
fusA_rv	GGAATGTTACCACCCTTCAC		
**RecA - DNA repair recombinase**			
recA_fw	GCTGCCATGGACAAGATAG	569	468
recA_rv	ACACCGATTTTCTCACGC		

Final length of concatenated MLST fragments: 3399bp

PCR protocol

All the primers have been designed with a GC content of 40-60% and a melting temperature (tm) of 54-56°C. The following PCR programs are suggestions and should be adjusted according to the specifications of the polymerase used.

Phusion polymerase

Initial denaturation: 98°C 30s
- Denaturation: 98°C 10s
- Annealing: 60°C 20 s
- Extension: 72°C 30s
- Repeat previous 3 steps 30 times

Final extension: 72°C 5m

Taq polymerase

Initial denaturation: 95°C 30s
- Denaturation: 95°C 10s
- Annealing: 55°C 20 s
- Extension: 72°C 30s
- Repeat previous 3 steps 30 times

Final extension: 72°C 5m

Table S2. Software programs and packages used in this study along with their versions and usage.

**Programs/packages**	**Version**	**Usage**
R	4.1.2	Basic automation and analysis
Rstudio	1.4.1106-5 - Build 372	R Integrated Development Environment
ClustalW	2.1	Multiple sequence alignment
Tidyverse	1.3.1	Increased R functionality
Shovill	1.0.9	WGS data assembly
Ubuntu	20.04	Running programs in Linux environment
FastQC	0.11.9	WGS data quality control
Prokka	1.14.5	Genome annotation
Roary	3.11.2	Pangenome assembly
Scoary	1.6.12	Pangenome analysis
Blast+	2.11.0	Gene identification
Expasy translate tool	-	Nucleotide to amino acid translation
Fasttree	2.1.11	Phylogenetic analysis
iTOL	6.5.8	Visualization of phylogenetic tress
goeBURST	1.2.1	Clonal complex identification
Grapetree	1.5.0	Visualization of ST geographical distribution
